# Ultrathin Multi‐Doped Molybdenum Oxide Nanodots as a Tunable Selective Biocatalyst

**DOI:** 10.1002/advs.202500643

**Published:** 2025-10-03

**Authors:** Bao Yue Zhang, Farjana Haque, Shwathy Ramesan, Sanjida Afrin, Muhammad Waqas Khan, Haibo Ding, Xin Zhou, Qijie Ma, Jiaru Zhang, Rui Ou, Md Mohiuddin, Enamul Haque, Yichao Wang, Azmira Jannat, Yumin Li, Robi S. Datta, Kate Fox, Guolang Li, Hujun Jia, Jian Zhen Ou

**Affiliations:** ^1^ School of Engineering RMIT University Melbourne Victoria 3000 Australia; ^2^ Florey Institute of Neuroscience and Mental Health Parkville Victoria 3052 Australia; ^3^ School of Science RMIT University Melbourne Victoria 3000 Australia; ^4^ State Key Laboratory of Digital Medical Engineering School of Biological Science and Medical Engineering Southeast University Nanjing 211189 China; ^5^ Department of Computer Science Information Technology Management Hong Kong Baptist University Hong Kong 999077 China; ^6^ School of Microelectronics Xidian University Xi'an 710071 China

**Keywords:** biocatalyst, cancer therapy, cytotoxicity, multi‐doped molybdenum oxide, reactive oxygen species

## Abstract

The reactive oxygen species (ROS) serve a significant role in cancer therapy due to their oxidative capabilities to modulate cellular functions from homeostasis to apoptosis. While conventional noble metal nanoparticles exhibit superior biocatalytic efficacy in ROS induction, their indistinctive toxicity toward cells and organisms limit their potential for targeted cancer therapy. Here, ultrathin biocompatiable molybdenum oxides (MoO_x_) nanodots are explored that simultaneously incorporate hydrogen (H^+^) and ammonia (NH_4_^+^) dopants, subsequently their electronic band structures can be modulated by both the relative contents of H^+^ and NH_4_^+^ dopants for efficient generation of ROS. An ultrafast and repeatable dye degredation capability in the absence of light is find in MoO_x_ doped with low H^+^ and high NH_4_^+^, in which hydroxyl radicals (·OH) is identified as the agent stimulating this ROS‐driven process through scavenger analysis. More importantly, the selective biocatalytic potential of such a multi‐doped MoO_x_ is demonstrated by the comprehensive assay analysis, revealing a three‐fold greater cytotoxicity toward HeLa cancer cells within 24 h compared with those of HEK293T healthy control. The finding shines a light on the targeted cancer therapies that spare healthy cells, showing the potential of multi‐doped metal oxide as a biocompatiable alternatives to noble metals in selective cytotoxicity against tumor cells.

## Graphical abstract

This paper presents an advanced multi‐doped molybdenum oxide nanodots with tunable ammonium and proton doping, exhibiting strong ROS generation. The material demonstrates ultrafast and complete dye degradation and unique selective toxicity toward cancer cells, highlighting its promising potential for cancer therapy applications.

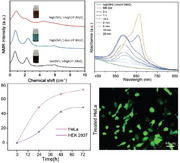

## 1 Introduction

ROS are highly reactive molecules that play crucial roles in various biological and environmental processes. It is also recognized as key player in the photodegradation of organic pollutants due to their strong redox properties.^[^ ^1^, ^2^ ^]^ In the field of biotechnology, as pervasive byproducts of oxygen metabolism, ROS significantly influence diverse cellular behaviors, such as signaling, metabolic functions, and tumorigenesis. Recent research has advanced our understanding of ROS, including their origin, types, levels, localization, and persistence, leading to a more comprehensive view of the complex roles ROS play in cancer therapies.^[^ ^1^ ^]^ Typically, ROS can enable cancer therapy through induction of oxidative stress,^[^ ^3^, ^4^ ^]^ direct deoxyribonucleic acid (DNA) damage,^[^ ^5^ ^]^ disruption of redox balances within cancer cells,^[^ ^6^ ^]^ and interference with cellular signaling pathways.^[^ ^7^ ^]^ Cancer cells usually express higher baseline levels of ROS compared to healthy cells and have a more imbalanced internal environment.^[^ ^8^ ^]^ This characteristic makes them more sensitive to external ROS levels, which can potentially be exploited for selective cancer therapies.

Inspired by natural enzymes, nanomaterials featuring structural vacancies and substitutional defects have been shown to exhibit highly effective biocatalytic activity, mimicking the performance of natural enzyme complexes.^[^ ^9^ ^]^ Recently, certain nanomaterials have been demonstrated to serve as an effective enzyme like compound to catalyst ROS with or without photo excitation, thanks to their favorable band diagram in reference to the redox potential of generating ROS.^[^ ^10^, ^11^, ^12^ ^]^ This demonstrates their potential as versatile tools in enhancing ROS‐mediated therapeutic strategies in cancer treatment. Metallic nanomaterials, dominated by noble metal nanoparticles (NPs), have demonstrated strong potential in the catalytic generation of ROS, in which the oxygen acts as an electron scavenger that inhibits fast recombination of the electron‐hole pairs, promoting the production of ROS.^[^ ^13^ ^]^ Some studies have attributed such a phenomenon to the localized surface plasmon resonance (LSPR), which is made up of collective oscillations of free charge carriers.^[^ ^13^ ^]^ However, intrinsic noble metal nanoparticles are known for their poor biocompatibility and strong toxicity due to their affinity for specific chemical bonds can disrupt biomedical processes, and lead to cellular dysfunction by releasing cytoplasmic contents. Besides, these nanoparticles tend to aggregate into larger particles which deteriorates their adverse effects and their catalytic efficiency.^[^ ^14^, ^15^ ^]^ Nonetheless, recent research further revealed the role of electronic structure and redox chemistry in the photogeneration of ROS by nanomaterials. The results indicate the electronic properties remain exclusive for metallic nanoparticles, which showcases the possibility of realizing efficient production of ROS using non‐noble metal NPs, such as engineered metal oxides.^[^ ^12^, ^16^, ^17^ ^]^

Metal oxides have shown significantly better biocompatibility with an enhanced affinity toward biosamples. Such characteristics possibly enable the selective catalysis of ROS targeting tumor cells. Degenerately doped metal oxides, heavily infused with external ions or functional groups, have been demonstrated to possess large amounts of free electrons, comparable to those found in noble metals.^[^ ^18^ ^]^ Additionally, their bandgaps are narrowed and generate strong localized plasmonic resonance similar to that of noble metals.^[^ ^19^ ^]^ These characteristics position them a promising and cost‐effective alternative to noble metal NPs. Molybdenum (Mo) oxides are amongst the most functional oxides in various applications due to their unique features in hosting dopant intercalation or atomic vacancies. For instance, Mo oxide readily accommodates various types of dopants, including H^+^, NH_4_^+^, Li^+^, etc. In the meantime, it can exhibit a wide range of substoichiometries such as MoO_x_ (2<x<3).^[^ ^18^ ^]^ This results in a transformation of the oxide's behaviors from semiconducting to metallic, which is contingent on the degree of atomic vacancy or dopant concentration. These inherent properties offer exceptional foundations for the effective utilization of these oxides in advanced systems.

Given the major toxicity mechanism of nanomaterials toward biomolecules is oxidative stress which is derived from the generated ROS, the Mo oxide needs to be engineered with a suitable electronic band energy to initiate ROS generation. Previous reports showed that the efficacy of singly doped Mo oxide (i.e., H^+^ or NH_4_^+^ doped MoO_x_) still mains limited ROS induction capability, possibly due to the constrained doping levels and unfavorable band structure.^[^ ^14^, ^20^ ^]^ Such insufficient toxicity toward tumor cells and high stability in biosamples impede their potential for tumor eradication and therapeutic interventions. It's worth noting that certain multi‐doped metal oxides are emerging as capable initiators of advanced oxidation processes for the complete degradation of organic dye.^[^ ^21^, ^22^ ^]^ This performance possibly benefits from the improved doping level enabled by multiple ions, which effectively mitigate the wide bandgap of metal oxides and consequently amplify the production of electron‐hole pairs. Despite these advancements, the realization of molybdenum oxide with multiple dopants remains less explored, leaving their potential to harness ROS for cancer ablation yet to be fully revealed.

In this work, we have developed an innovative one‐step approach to create multi‐doped Mo oxide nanodots, with varying doping proportions achieved by adjusting the quantity of Dimethylformamide (DMF) solvent used during the synthesis process. Among the three multi‐doped MoO_x_ created, the multi‐doped MoO_x_ produced with the low H^+^ and high NH_4_^+^ doping exhibited ultrafast catalytic activity, as evidenced by its rapid degradation of methylene blue (MB) within 20 min even in the absence of light. Furthermore, employing three distinct scavengers allowed us to discern the ·OH as the agent responsible for the degradation of dye. To thoroughly investigate the selective cytotoxic attributes and the underlying mechanism, we performed 3‐ (4,5‐dimethylthiazol‐2‐yl)‐2,5‐diphenyltetrazolium bromide (MTTon studies, measurements of mitochondrial membrane potential, and assessments of nuclear morphological changes on both the cervical cancer cell line, HeLa, and a normal cell line (HEK293T). In addition, an annexin assay was utilized to verify the cell death induced by exposure to multi‐doped Mo oxide nanodots. As a result, this research explored the cytotoxic impact of multi‐doped MoO_x_ nanodots on invasive cancer cell lines only, highlighting their ability to selectively induce apoptosis in tumor cells but to maintain healthy cell functions. This discovery underlines the promising selective biocatalyst potential of the multi‐doped Mo oxide, permitting its viability for future application in cancer therapy.

## 2 Results and Discussion

### 2.1 Synthesis of NH_4_^+^ and H^+^ Doped MoO_x_

Ammonium molybdate, (NH_4_)_2_MoO_4_, is a widely employed precursor for the synthesis of MoO_x_ ‐based materials via hydrothermal methods. In this work, three multi‐doped MoO_x_ was made using ammonium molybdate, hydrazine powder in a mixed solvent of water and DMF, in which the ammonium molybdate and water serve as an active NH_4_^+^ and H^+^ dopant sources, respectively. Additionally, hydrazine acts as an electron donor, creating a reducing environment.^[^ ^23^ ^]^ In brief, (NH_4_)_2_MoO_4_ and NH_2_NH_2_ were first dissolved in the three DMF‐water solutions under stirring with the DMF ratio of 20%, 80%, and 100%, respectively. The mixtures were then transferred into Teflon‐lined stainless‐steel autoclaves for hydrothermal synthesis of the final product of three N and H doped MoO_x_ samples. During the reaction, elevated temperature and pressure likely promote the decomposition of ammonium molybdate into reduced MoO_x_ compounds, releasing NH_3_.^[^ ^24^, ^25^ ^]^ The dissolved NH_3_ forms NH_4_^+^ ions, which incorporate into the MoO_x_ lattice, resulting in an “ammonium molybdenum bronze” via a redox‐driven mechanism.^[^ ^26^, ^27^ ^]^ For proton intercalation, water serves as the H⁺ source.^[^ ^28^ ^]^ In the presence of a strong reductant like hydrazine, water can dissociate on the oxide surface, generating acidic O–H protons that facilitate hydrogen molybdenum bronze formation.^[^ ^18^, ^26^, ^28^, ^29^, ^30^ ^]^ This process involves hydrazine reducing Mo^6+^ to lower oxidation states such as Mo^5+^ and Mo^4+^, which in turn induces the formation of oxygen vacancies within the MoO_3_ lattice. These vacancies act as active sites for water adsorption and dissociation, enabling proton transfer to adjacent lattice oxygen atoms and stabilizing the intercalated protons through O─H bond within the MoO_3_ structure.^[^ ^31^ ^]^ Given the rise to the proton‐doped phase, this is termed hydrogen molybdenum bronze (H_x_MoO_3_), as widely reported in literature.^[^ ^18^, ^30^, ^32^, ^33^ ^]^ In this work, the proton intercalation was confirmed by ^1^H NMR spectroscopy, which reveals the presence of lattice‐stabilized protons in our multi‐doped MoO_x_ samples. This mechanistic pathway highlights the role of hydrazine not only in Mo reduction and in promoting proton incorporation through water dissociation.

**Figure 1:**
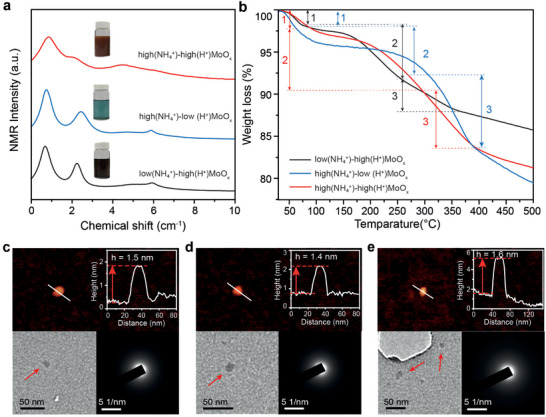
a) ^1^H MAS NMR spectra with the samples optical image (inset from top to bottom), with the inset showing the original color, and b) TGA spectra of low(NH_4_^+^)‐high(H^+^)MoO_x_, high(NH_4_^+^)‐low(H^+^)MoO_x_, high(NH_4_^+^)‐high(H^+^)MoO_x_. Top: AFM imaging (left) and height profile (right), bottom: TEM imaging (left) and SAED patterns (right) of c) low(NH_4_^+^)‐high(H^+^)MoO_x_, d) high(NH_4_^+^)‐low(H^+^)MoO_x_, and e) high(NH_4_^+^)‐high(H^+^)MoO_x_.

The three materials display unique colors of red, blue and black, upon the different amounts of DMF applied (**Figure** **1**a). Given that color alterations in doped MoO_x_ samples often signify changes in the doping levels, it is posited that the dopant concentrations of N and H in MoO_x_ can be adeptly modulated by varying the composition of the precursor solution.

In order to confirm the doping of H^+^ and NH_4_^+^ during the hydrothermal synthesis, which aligns with our hypothesis, we employed solid‐state ^1^H magic‐angle spinning (MAS) nuclear magnetic resonance (NMR) spectroscopy. As shown in Figure 1a, the presence of peaks at 0.8 ppm across all three samples suggests the existence of OH bonds, presumably indicative of molybdenum hydroxide (Mo‐OH) groups. These groups predominantly bonded with asymmetric oxygen atoms (corner‐sharing oxygen) and with terminal oxygen atoms (unshared oxygen atoms).^[^ ^30^ ^]^ For the sample synthesized in 20% DMF solution, the peak at 2.6 ppm exhibits broadening, while the peak at 4.2 ppm becomes more pronounced, suggesting an increased H^+^ doping level.^[^ ^18^ ^]^ The indication is that the terminal oxygen atom sites within multi‐doped MoO_x_ primarily serve as the preferential location for a large amount of ion dopants.^[^ ^30^, ^34^, ^35^, ^36^ ^]^ Therefore, we reasonably associate the emergence of the peak at 2.6 ppm to H^+^ bonded to the asymmetric coordinated oxygen atoms, while the 4.2 ppm for the sample synthesized in 20% DMF can be attributed to and H^+^ bonded with the terminal oxygen atoms.^[^ ^18^ ^]^ The peak found at the 5.8 ppm for the samples prepared in 80% and 100% DMF could be attributed to the protons at the N‐atom, indicates the presence of NH_4_^+^ in the samples.^[^ ^37^ ^]^ For all the samples, a small hump can be observed in the region between 0.5 and 1 ppm, which can be ascribed to surface adsorbed water molecules that possibly originate from the ambient environment.^[^ ^18^ ^]^ In the meantime, the amount of H^+^ and NH_4_^+^ dopants can be further determined through thermogravimetric analysis (TGA) operated under an N_2_ gas environment. As shown in the Figure 1b, the TGA curves could be divided into three main steps. The initial weight loss of the sample synthesized in 20% DMF at 30–67 °C is 2.23% which was followed by a 7.31% decrease in mass at 67–330 °C and a further 6.89% decrease at 330–393 °C. Here, the preliminary weight loss, which happened below 100 °C could be attributed to the removal of the surface adsorbed water molecules^[^ ^38^ ^]^ whereas the next two stages were due to the release of the hydrogen dopant in the form of crystalline water molecules and nitrogen dopant as NH_4_^+^ ions.^[^ ^39^, ^40^, ^41^ ^]^ On the other hand, in the case of the sample synthesized in 80% DMF, the second and third steps stayed at a slightly lower temperature which reveals a slight increase and decrease in the H^+^ and NH_4_^+^ dopant levels. However, the TGA behavior of the sample synthesized in 100% DMF was different from the other two samples with the steps stopped at a lower temperature of below 368 °C and demonstrating the highest H^+^ and lowest NH_4_^+^ dopant fractions in the structure. Based on the TGA weight loss of the material, we are able to estimate the amount of H^+^ and NH_4_^+^ in the three samples. As shown in Table S1 (Supporting Information), the doped MoO_x_ synthesized using 100%, 80%, and 20% DMF solution are calculated to be (NH_4_)_0.36_H_1.42_MoO_3,_ (NH_4_)_0.81_H_1.1_MoO_3_, and (NH_4_)_0.66_H_1.4_MoO_3_, respectively. The variation of doping concentration could be derived by DMF solvent addition. Although DMF does not directly act as a dopant source, its strong hydrogen bonding with water may influence proton generation and doping.^[^ ^42^ ^]^ In the 20% DMF solvent, the lower DMF content allows water to dominate the solvation environment, promoting greater H^+^ generation and doping. At 80% DMF, the increased DMF clearly reduces water activity, leading to a lower level of H^+^ doped into MoO_3_, while the NH_4_^+^content does not show a significant change. Notably, we observed a decrease in total dopant content (0.81 NH_4_^+^ and 1.1 H^+^, totaling 1.91 ionic dopants). In the 100% DMF case, a significant drop in NH_4_^+^ content was observed, possibly freeing more space for proton incorporation. However, due to the further reduced availability of free water, the overall dopant level decreased further (0.36 NH_4_^+^ and 1.42 H^+^, totaling 1.78 ionic dopants) (Table S1, Supporting Information). Overall, the increased amount of DMF caused reduced dopant amount, but the precise mechanism by which DMF influences the balance between NH_4_^+^and H^+^ doping remains to be further investigated.^[^ ^42^ ^]^ This elevated H⁺ content likely enhanced interactions with the MoO_3_ lattice, ultimately leading to the lowest NH_4_^+^ doping among the three samples. The elemental composition of all three doped MoO_x_ synthesized using 100%, 80%, and 20% DMF solution are calculated to be (NH_4_)_0.36_H_1.42_MoO_3_, (NH_4_)_0.81_H_1.1_MoO_3_, and (NH_4_)_0.66_H_1.4_MoO_3_, respectively. The three samples are named according to their H and N doping levels as “low(NH_4_^+^)‐high(H^+^)MoO_x_”, “high(NH_4_^+^)‐low(H^+^)MoO_x_” and “high(NH_4_^+^)‐high(H^+^)MoO_x_”, respectively

Atomic force microscopy (AFM) was employed to conduct the morphological characterization of the respective samples. As shown in a representative AFM imaging in Figure 1c–e, three types of multi‐doped MoO_x_ samples upon different doping levels depict similar nanodot with a lateral dimension of <40 nm and a thickness of ≈1.5 nm, representing a single unit cell thick MoO_x_.^[^ ^43^ ^]^ Large area images with more sample nanodots are shown in Figure S1 (Supporting Information). To further determine the average dimensions of the materials, histogram plots with normalized distribution curves were generated by measuring 30 samples from AFM images. The average thickness of the multi‐doped MoO_x_ was found to be ≈1–2 nm (Figure S1, Supporting Information). Transmission electron microscopy (TEM) was further carried out to evaluate the dimensions and crystal structure of the sample. The images of samples in Figures 1c–e and S1 (Supporting Information) are found to be well‐matched with the AFM results, in which the corresponding selected area diffraction (SAED) patterns illustrate the amorphous nature of all three multi‐doped MoO_x_ samples.

**Figure 2:**
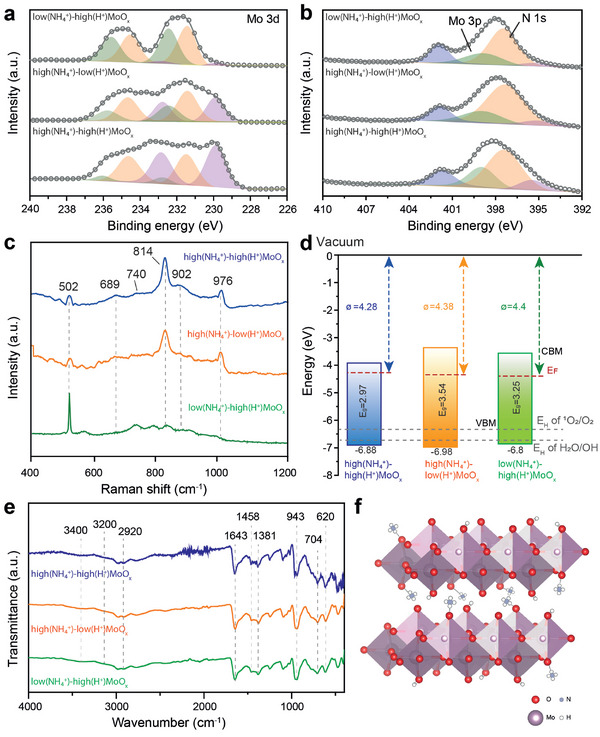
a) Mo3d XPS spectra, b) N1s XPS spectra overlapped with Mo3p, c) Raman spectra, d) band structure, and e) FTIR spectra of high(NH_4_^+^)‐high(H^+^)MoO_x_, high (NH_4_^+^)‐low(H^+^)MoO_x_, and low(NH_4_^+^)‐high(H^+^)MoO_x_. f) Illustration of multi‐doped MoO_x_.

To further confirm the chemical composition of the three types of multi‐doped MoO_x_ samples, X‐ray photoelectron spectroscopy (XPS) measurements were conducted to reveal the doping levels of H^+^ and NH_4_^+^. The peaks in **Figure** **2**a stand for the Mo3d spectra, in which the peak deconvolution divulges the contribution from Mo3d. The doublet resonances observed at 236.0 and 232.8 eV can be respectively correlated with the Mo3d 3/2 and Mo3d 5/2 orbital electrons of Mo^6+^. In each of the three types of multi‐doped MoO_x_ samples, there is an intercalation of H^+^ and NH_4_^+^ and the simultaneous introduction of charge carriers leads to the appearance of substoichiometric Mo^5+^ and Mo^4+^. Resonance peaks at 231.6 and 234.8 eV are indicative of the Mo^5+^ oxidation state, whereas the Mo^4+^ state is suggested by peaks identified at 230.5 and 233.8 eV.^[^ ^18^ ^]^ In the context of the low(NH_4_^+^)‐high(H^+^)MoO_x_ sample, the Mo3d signature primarily exhibits a high concentration of Mo^5+^ and Mo^6+^, with the Mo^6+:^ Mo^5+:^ Mo^4+^ ratio calculated to be 44: 52: 4. The amount of Mo^4+^ is observed to significantly increased in the moderately doped 80% sample with the smaller Mo^6+:^ Mo^5+:^ Mo^4+^ ratio of 36: 38: 26. In the context of high(NH_4_^+^)‐high(H^+^)MoO_x_, the signature of Mo^6+^ almost drops to the half of that in low(NH_4_^+^)‐high(H^+^)MoO_x_, and instead, Mo3d primarily exhibits Mo^5+^ and Mo^4+^ oxidation states. The calculated distribution ratio of Mo^6+^, Mo^5+^ and Mo^4+^ in this sample is determined to be 26: 32: 42. The results comply with the most weight loss of the observation in high(NH_4_^+^)‐high(H^+^)MoO_x_. The NH_4_^+^ doping was verified through the N1s spectrum measurement. As is known, N1s overlapped with the band energy with Mo3p in molybdenum oxide.^[^ ^26^ ^]^ According to Figure 2b, the peak centered at 397.1 eV can be attributed to the N in the form of NH_4_^+^,^[^ ^26^ ^]^ which corresponds to a lower Mo phase with the N coordination. While the peak at 398.8 eV represents Mo^6+^.^[^ ^26^ ^]^ The weak peak appearing at lower band energy of 395.5 eV is attributed to Mo^4+^. The shake‐up satellite peak at a higher band energy of 401.5 eV can be ascribed as the surface absorbed nitrogen.^[^ ^26^ ^]^ The peak at 395.5 evident the presence of Mo^4+^ and this alignment suggests a reduction in the Mo phase with the N coordination, signifies that as N doping increases across the three samples, the valence state of the multi‐doped MoO_x_ progressively diminishes.^[^ ^26^ ^]^ The gradually increased amount of Mo^4+^ and N have been found in an order of low(NH_4_^+^)‐high(H^+^)MoO_x_ < high(NH_4_^+^)‐low(H^+^)MoO_x_ < high(NH_4_^+^)‐high(H^+^)MoO_x_. The ratio of nitrogen is calculated from the N1s 397.1 eV peak. The highest amount of N doping was found in high(NH_4_^+^)‐low(H^+^)MoO_x_, which matches well with the conclusion from TGA characterization. XPS O1s spectra of three samples are shown in Figure S2 (Supporting Information). For all three multi‐doped MoO_x_ samples, the peak at 530.8 eV can be attributed to the Mo^6+^‐O group.^[^ ^18^ ^]^ A relatively subdued peak, identified at 531.9 eV can be ascribed to the presence of adsorbed OH groups and the formation of Mo^5+^─O bond.^[^ ^44^ ^]^ An additional peak identified at 532.8 eV is attributed to the Mo^4+^─O bond.^[^ ^26^ ^]^ The observation is consistent with the TGA result that high(NH_4_^+^)‐high(H^+^)MoO_x_ holds the largest amount of dopant which leads to the more substoichiometric phase of Mo among all three samples.

Raman spectroscopy was utilized to analyze the three variants of multi‐doped MoO_x_ samples, with the aim of revealing the influence of dopants on the crystal alteration and atom bonding (Figure 2c). In the case of high(NH_4_^+^)‐high(H^+^)MoO_x_ and high (NH_4_^+^)‐low(H^+^)MoO_x_, the distinguished peaks at 814 cm^−1^ can be attributed to the stretching mode of Mo─O─Mo from the MoO_6_ octahedra.^[^ ^26^ ^]^ A strong peak at 502 cm^−1^, which can be assigned as an O─Mo─O bonding vibration, was observed in low(NH_4_^+^)‐high(H^+^)MoO_x_ but appeared weak in the other two samples.^[^ ^26^ ^]^ Other two peaks at 976 and 740 cm^−1^ are attributed to the Mo^5+/4+^─O stretching vibration, demonstrating the presence of NH_4_^+^ ions in the materials.^[^ ^26^ ^]^ A weak peak appeared at 689 cm^−1^ in high(NH_4_^+^)‐high(H^+^)and high (NH_4_^+^)‐low(H^+^)MoO_x_ but was not shown in low(NH_4_^+^)‐high(H^+^)MoO_x_ indicates the O─Mo─O bond.^[^ ^45^ ^]^

In order to explore the electron band structure of three multi‐doped MoO_x_ samples, XPS valence spectra were obtained to reveal the position of the valence band. As shown in Figure S3 (Supporting Information), for the high(NH_4_^+^)‐high(H^+^)MoO_x_, high(NH_4_^+^)‐low(H^+^)MoO_x_, and low(NH_4_^+^)‐high(H^+^)MoO_x_ exhibit a valence band of 2.6, 2.5, and 2.3 eV away from the Fermi level. A weak hump between the Fermi level and edge of valence band, which corresponds to the free electron occupation in Mo 4d state, can be observed from these samples, indicating a slight metallic feature of the doped MoO_x_ samples.^[^ ^18^ ^]^ Considering the unit cell of typical MoO_3_ crystal (Space group *Pnma*), and each NH_4_^+^ and H^+^ will contribute one free electron to the lattice, the free charge concentration can be calculated. According to the calculated chemical composition of three multi‐doped MoO_x_ in Table S1 (Supporting Information), the free charge carrier concentrations are calculated as 3.51 × 10^22^, 3.77 × 10^22^, and 4.02 × 10^22^ cm^−3^ for low(NH_4_^+^)‐high(H^+^)MoO_x_, high(NH_4_^+^)‐low(H^+^)MoO_x,_ and high(NH_4_^+^)‐high(H^+^)MoO_x_, respectively (Note S1, Supporting Information). In the meantime, Mott‐Schottky measurement was utilized to ascertain the location of the Fermi level. To determine the band structure, UV–vis measurement was conducted to extract the Tauc plot to characterize the optical bandgap property of the three doped MoO_x_. Assuming the defective MoO_x_ material is direct bandgap semiconductor,^[^ ^46^ ^]^ as shown in Figure S4 (Supporting Information), the high(NH_4_^+^)‐high(H^+^)MoO_x_, high(NH_4_^+^)‐low(H^+^)MoO_x_ and low(NH_4_^+^)‐high(H^+^)MoO_x_ show bandgaps of 2.97, 3.54, and 3.25 eV, respectively. According to Figure S5 (Supporting Information), the Mott‐Schottky plots demonstrates similar flat‐band potentials of ‐4.28, ‐4.38, and ‐4.4 eV in reference to vacuum for the three samples, respectively. Collectively considering the XPS valence band, optical bandgap measured, and the fermi location determined by Mott‐Schottky plots, the band structures of all three multi‐doped MoO_x_ samples can be depicted as Figure 2d.

To further confirm the presence of dopant groups in the three samples, Fourier transform infrared (FTIR) spectroscopic analyses were also performed in the 400–4000 cm^−1^ region. In Figure 2e, the weak IR bands identified at ≈3200 and 1458 cm^−1^ in all three samples are ascribed to the stretching and bending vibrations of the N─H bond respectively.^[^ ^47^, ^48^ ^]^ These two peaks as well as the ‐OH peak at ≈2920 cm^−1^, which is representing the presence of the crystal water in the sample, confirm the presence of H^+^ and NH_4_^+^ dopants in the crystal structure.^[^ ^49^ ^]^ Besides, the peaks at 3400 and 1643 cm^−1^ are ascribed to the O─H groups as well as the bending vibrations of the H‐O‐H from the water molecules, respectively.^[^ ^50^ ^]^ Another peak at 1381 cm^−1^ emerges due to the vibration of Mo‐OH groups, possibly originating from the bonds between H^+^ ions and the MoO_6_ octahedra as well as crystalline water. Moreover, the peak found at 620 cm^−1^ is a characteristic peak of the Mo─O vibration of the h‐MoO_3_ material, which is previously mentioned by Chithambararaj et al.^[^ ^47^ ^]^ Finally, the peaks at 943 and 704 cm^−1^ demonstrate the Mo = O characteristic stretching vibration and Mo‐O‐Mo asymmetric stretching mode of the MoO_x_ molecules respectively.^[^ ^47^ ^]^ The illustration of the multidoped MoO_x_ material is shown in Figure 2f.

### 2.2 Room Temperature Decomposition of Dye in Dark

**Figure 3:**
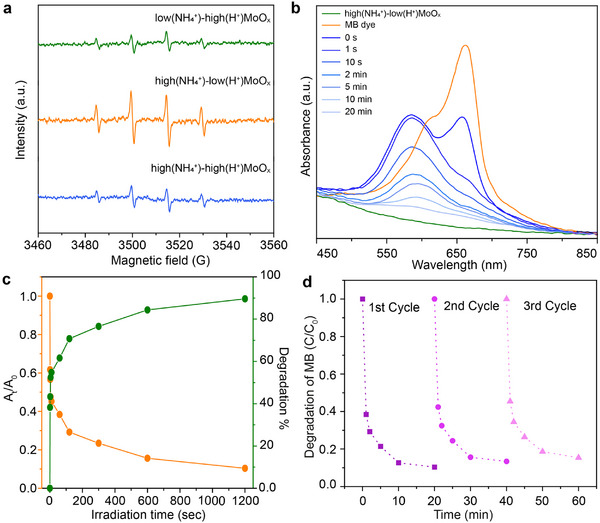
a) Electron paramagnetic resonance (EPR) signals for free radical trapping of ·OH for three multi‐doped MoO_x_. b) The UV–vis absorption spectrum of MB dye in the presence of the 2D high(NH_4_^+^)‐low(H^+^)MoO_x_ catalyst evaluated at room temperature and in a dark environment. c) Degradation of MB in the presence of the 2D high(NH_4_^+^)‐low(H^+^)MoO_x_ catalyst under room temperature and in dark environment. The normalised concentration of the MB observed at the 665 nm wavelength as a function of simulated solar light irradiation time. The degree of MB degradation was determined by calculating the variation of concentration (C/C_0_) based on the change in absorbance at 665 nm. d) Repeated cycles of the dye degradation in the dark.

To investigate the catalytic behavior in these multi‐doped MoO_x_ NPs, the free radical trapping and electron states were analysed by the electron paramagnetic pellisresonance (EPR) in dark. As shown in **Figure** **3**a, weak signals can be detected in high(NH_4_^+^)‐high(H^+^)MoO_x_ and low(NH_4_^+^)‐high(H^+^)MoO_x_ using DMPO‐·OH adducts, implying small amount of ·OH generation. While the high(NH_4_^+^)‐low(H^+^)MoO_x_ sample exhibited the much stronger signal, demonstrating large amount of ·OH production and potentially enhanced catalytic behavior. This can be attributed to the band structure alignment, where the redox potential of the H_2_O/·OH couple (≈6.7 eV vs vacuum, pH = 7) lies below the valence band maximum (VBM) of the multi‐doped MoO_x_ (Figure 2d), confirming that the oxidative potential of the generated holes is sufficient to drive ·OH formation.^[^ ^17^ ^]^ Moreover, the co‐doping with NH_4_^+^ and H^+^ likely introduces structural distortions and mixed‐valence Mo centers (e.g., Mo^6+^/Mo^5+^), which facilitate internal redox activity and spontaneous hole formation under dark conditions, further promoting ROS generation.^[^ ^51^ ^]^ While, the EPR signal for the TEMP – ^1^O_2_ adducts cannot be detected (Figure S6, Supporting Information), though the redox potential (E_H_) for ^1^O_2_/O_2_ (6.38 eV in reference to vacuum) is also lower than that of the doped MoO_x_, possibly because the oxidizing power is favorable to produce ·OH radicals in the material suspension.^[^ ^12^ ^]^ The high(NH_4_^+^)‐low(H^+^)MoO_x_ is selected as an representative sample to assess the surface activity and catalytic performances of all 2D multi‐doped MoO_x_ through the decomposition of an organic pollutant‐methylene blue (MB), which has been widely used as a standard dye in prior studies exploring photocatalytic activities.^[^ ^43^ ^]^ The multi‐doped MoO_x_ sample did not present any optical absorption at the wavelengths where MB dye has a strong absorption (Figure 3b). An investigation was conducted into the ultrafast degradation of the characteristic absorbance spectrum for MB dye facilitated by the catalyst of multi‐doped MoO_x_ nanodots under dark circumstances. Degradation of MB was performed and monitored over 20 min with data collected from the onset of mixing MB with the catalyst. The degradation ratio is determined by the intensity of the MB absorbance peak. Figure 3b shows the MB degradation in the presence of high(NH_4_^+^)‐low(H^+^)MoO_x_. The characteristic absorption peak of MB is centered at 610 and 660 nm and the catalyst did not present any absorption at this range. While just after seconds, the initial peak at 660 nm intensity dropped significantly. The total intensity of the main peak at 610 nm decreased by 50% after ≈10 s, and vanished in ≈20 min. The color change of MB indicates the breakdown of its chromophore configuration. Based on the degradation of the dye, the relative change in absorbance (A_t_/A_0_) and the percentage of dye degradation were calculated. As shown in Figure 3c, upon the irradiation time increases, under the presence of 2D high(NH_4_^+^)‐low(H^+^)MoO_x_ catalyst, 90% MB was degraded within 20 min. In comparison, both low(NH_4_^+^)‐high(H^+^)MoO_x_ and high(NH_4_^+^)‐high(H^+^)MoO_x_ exhibited inferior performance as a catalyst, where only 59% and 56% of MB were degraded respectively (Figure S7, Supporting Information). Furthermore, high(NH_4_^+^)‐low(H^+^)MoO_x_ was used to test for MB degradation three times with a duration of 20 min for each cycle (Figure 3d). The results indicate high(NH_4_^+^)‐low(H^+^)MoO_x_ catalytic performance is highly stable with little variance between each test.

To further demonstrate the strong catalytic performance of this multi‐doped MoO_x_ NPs, the dye degradation in dark performance of commercial MoO_3_, H⁺‐doped MoO_3_, NH_4_^+^‐doped MoO_3_ samples was systematically evaluated, with the results presented in Figure S8 (Supporting Information). The single doped MoO_3_ samples were fabricated following the method as reported.^[^ ^18^, ^26^ ^]^ The commercial and singly doped MoO_3_ samples showed minimal activity, achieving less than 3% dye removal within 20 min. In contrast, the multi‐doped MoO_x_ samples demonstrated significantly enhanced catalytic performance, achieving dye degradation efficiencies between 56% and 90%. This superior activity is attributed to the generation of a high concentration of ROS, particularly ·OH radicals, as confirmed by EPR analysis.

Metal oxides are widely reported for their photocatalytic performance, but mostly show an unstable result, which is often resulted from the strong surface aggregation of nanodots.^[^ ^52^, ^53^ ^]^ Therefore, the Zeta potential measurement was carried out to investigate the aggregation behavior for all three multi‐doped MoO_x_ nanodots after the photocatalytic process. The high(NH_4_^+^)‐high(H^+^)MoO_x_ and low(NH_4_^+^)‐high(H^+^)MoO_x_ nanodots have shown an apparently different Zeta potential of 32.2 and 25.8 mV, respectively. However, high (NH_4_^+^)‐low(H^+^)MoO_x_ nanodots exhibited a Zeta potential of 45.6 mV which signifies the much better dispersibility stability of this sample compared to that of the other two samples and possibly promoted the high‐quality photocatalytic performance (Figure S9, Supporting Information).

### 2.3 Selective Cytotoxicity

Among all types of ROS, hydroxyl radicals is the highly detrimental to cells since it can react across a wide range of macromolecules, including carbohydrates, nucleic acids, lipids, and amino acids.^[^ ^12^ ^]^ The bactericidal activities of metal oxides have been extensively investigated, and this phenomenon is commonly attributed to the degradation of plasma membranes by reactive oxygen species (ROS), particularly ·OH. This process results in the peroxidation of phospholipids and ultimately leads to cell death. Hence, the cytotoxicity facilitated by nanomaterials could potentially be harnessed for the targeted elimination of tumor cells.

**Figure 4:**
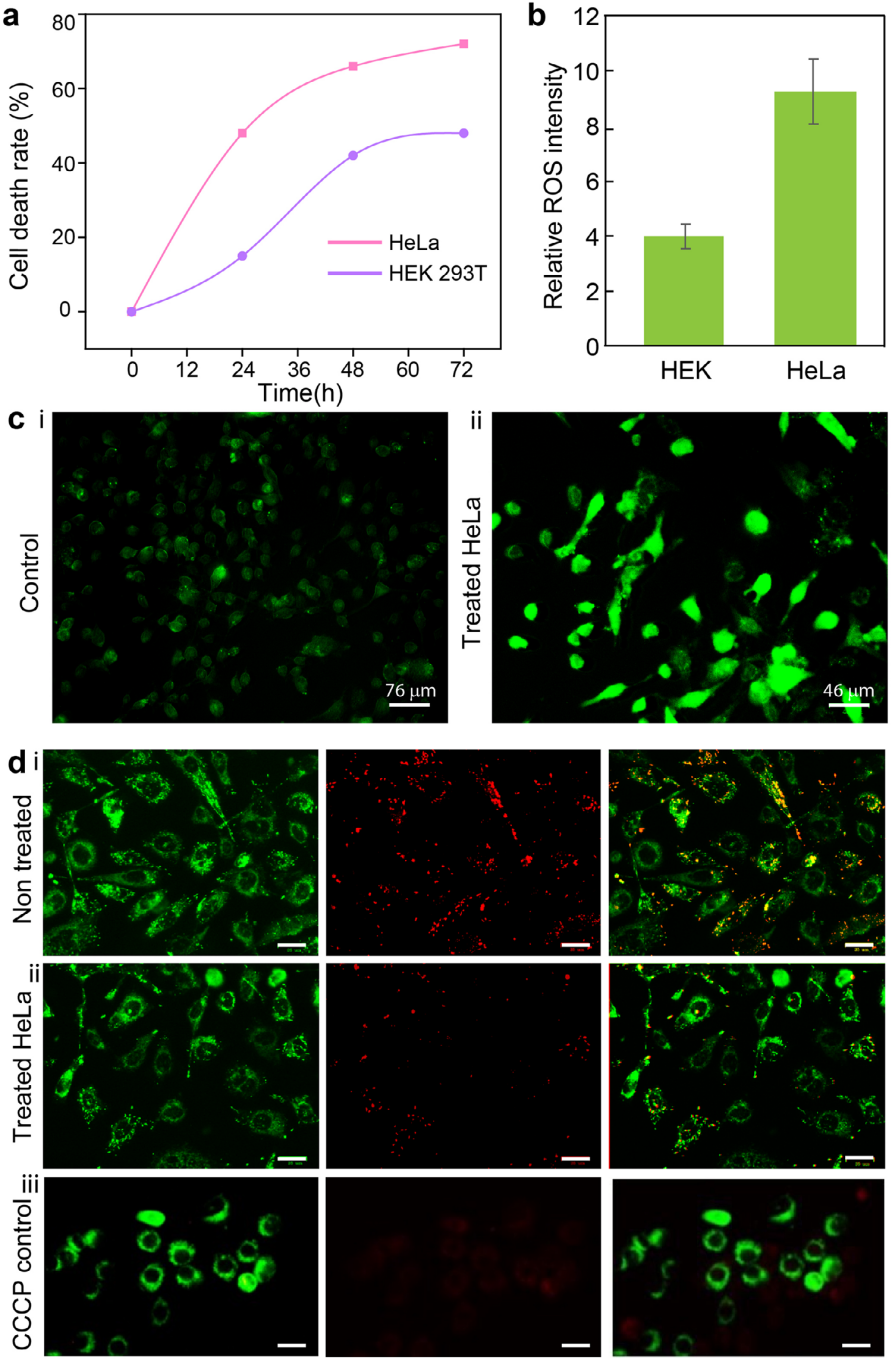
a) Cytotoxicity of high(NH_4_^+^)‐low(H^+^)MoO_x_ nanodot toward HEK 293T and HeLa cells. Cells were treated with MoO_x_ nanodots for 24, 48, and 72 h at 375 µg mL^−1^ concentration to observe the percentage of cell death. Data are shown as means ± standard deviation (S.D.) b) Quantitative determination of ROS was accomplished using fluorometric techniques, in the presence of DCF‐DA and conducting measurements at an excitation wavelength of 485 nm and an emission single wavelength of 535 nm. The control cells represent untreated cells while multi‐doped MoO_x_ data represented the cells treated with multi‐doped MoO_x_ nanodots at its IC_50_ concentration. c) Qualitative analysis of the ROS formed by the treatment of cells with DCF‐DA. The brighter treated cells represent higher ROS levels formed due to the induction of apoptosis by the MoO_x_ nanodots. d) MMP image showing i. orange‐green spindle structures in the control group (untreated cells); ii. increased green aggregation in the high (NH_4_^+^)‐low(H^+^)MoO_x_ treated cell group indicating depolarization and mitochondrial collapse; (iii) CCCP treated HeLa cell as a positive control, showing enhanced green aggregation and indicating gradual destruction of living cells. Scale bards: 20 µm.

The high(NH_4_^+^)‐low(H^+^)MoO_x_ was tested for its selective cytotoxicity properties while the commercial silver NPs were used as a control sample. The high(NH_4_^+^)‐low(H^+^)MoO_x_ nanodots were tested at concentrations ranging from 700 to 5.47 µg mL^−1^, while the silver NPs were tested at concentrations ranging from 350 to 0.7 µg mL^−1^. The cervical cancer cell line (HeLa cells) and a healthy cell line (HEK293T) were used as experimental and control groups, respectively. The cells were exposed to the nanodots for different durations, and measurements were taken at 24, 48, and 72 h post‐exposure. As seen in **Figure** **4**a, high(NH_4_^+^)‐low(H^+^)MoO_x_ nanodots have induced ≈50% cell death in HeLa cells and only 18% cell death in HEK293T cells after 24 h of exposure. This observation indicates a selective and higher cytotoxic effect of the nanodots toward the HeLa cells compared to the healthy counterpart. Over time, the viability rates of both normal and cancer cell lines followed similar trends. After 72 h of exposure to high(NH_4_^+^)‐low(H^+^)MoO_x_, both cell types reached a plateau in viability rates, indicating a saturation point. In addition, the MTT assay was performed to evaluate the viability rates of both HeLa and HEK293T cell lines after 24 h of exposure to silver NPs at various concentrations. First, the two types of cells were treated with silver NPs to observe the specificity of the cancer cell lines. The half inhibitory concentrations (IC_50_) of silver NPs against both cell lines were determined to be ≈25 µg mL^−1^ as shown in Figure S10 (Supporting Information). The cell death rates of both normal and cancer cell lines were both very high and comparable, reaching up to 90% at the concentration of 350 µg mL^−1^, indicating that silver NPs did not exhibit selective cytotoxicity toward cancer cell lines. While the IC_50_ of high(NH_4_^+^)‐low(H^+^)MoO_x_ toward HeLa cell line was determined to be ≈350 µg mL^−1^ as shown in Figure S11 (Supporting Information). Even at this high concentration, high(NH_4_^+^)‐low(H^+^)MoO_x_ only presented mild cytotoxicity toward the healthy counterpart – HEK 293T cell line with a cell death rate of ≈18%. Furthermore, the MTT assay analysis was conducted on both cell lines after exposing high(NH_4_^+^)‐low(H^+^)MoO_x_ for additional time up to 48 h and 72 h, respectively. Figure S12 (Supporting Information) shows that the cell death rate would gradually increase upon longer exposure time to high(NH_4_^+^)‐low(H^+^)MoO_x_, but still with clear stronger cytotoxicity against HeLa cells compared to HEK 293T cells at the IC_50_ concentration. Though the high(NH_4_^+^)‐low(H^+^)MoO_x_ shows slightly higher IC_50_ value compared to that of the silver NPs, the results demonstrated a high specificity in cytotoxicity, with high(NH_4_^+^)‐low(H^+^)MoO_x_ nanodots inducing a significantly higher cell death rate in HeLa cells compared to HEK293T cells, especially within the first 24 h. The overall outcome suggests distinct cytotoxicity in tumor and healthy cells from the multi‐doped MoO_x_ nanodots.

A 2D viability assay was utilized to study cytotoxicity by measuring the ROS formed due to the presence of the multi‐doped MoO_x_ nanodots. Figure 4b,c shows the quantitative and qualitative measurements of the ROS levels in cells post their exposure to the synthesized multi‐doped MoO_x_. A 2.5‐fold increase in ROS levels was observed in HeLa cells when exposed to the high(NH_4_^+^)‐low(H^+^)MoO_x_ at its IC_50_ concentration. The increased intensity of the green fluorescence obtained due to the deacetylation of the non‐fluorescent dichlorodihydrofluorescein (DCF)‐diacetate (DA) to a fluorescent DCF in the presence of increased ROS levels is evident by the higher green fluorescence observed in the high(NH_4_^+^)‐low(H^+^)MoO_x_‐treated HeLa cells (Figure 4c‐i), compared to the control HEK293T cell group (Figure 4c‐ii).

The antiproliferative activity of the multi‐doped MoO_x_ nanodots was investigated by assessing the mitochondrial membrane potential (MMP) with the aid of JC‐1 staining 24 h after the cells were treated with the high(NH_4_^+^)‐low(H^+^)MoO_x_. As seen in Figure 4d‐i, the polarized mitochondrial spindle‐like structure marked by punctate orange‐red fluorescent staining is clearly visible in the control group. The treated cells; on the other hand displayed the signs of depolarization observed by the loss of orange‐green aggregates being replaced by bright green staining (Figure 4d‐ii), indicating a mitochondrial membrane collapse and hence the induction of apoptosis by multi‐doped MoO_x_ material. The image of cells treated with chlorophenyl hydrazone (CCCP) as a positive control shows strong green fluorescence, indicating JC‐1 monomers in depolarised mitochondria (Figure 4d‐iii). The middle panel shows minimal red fluorescence, consistent with the loss of JC‐1 aggregates due to mitochondrial dysfunction. The comparative results demonstrate the effectiveness of the multi‐doped MoO_x_ in inducing selective toxicity toward cancer cells.

**Figure 5:**
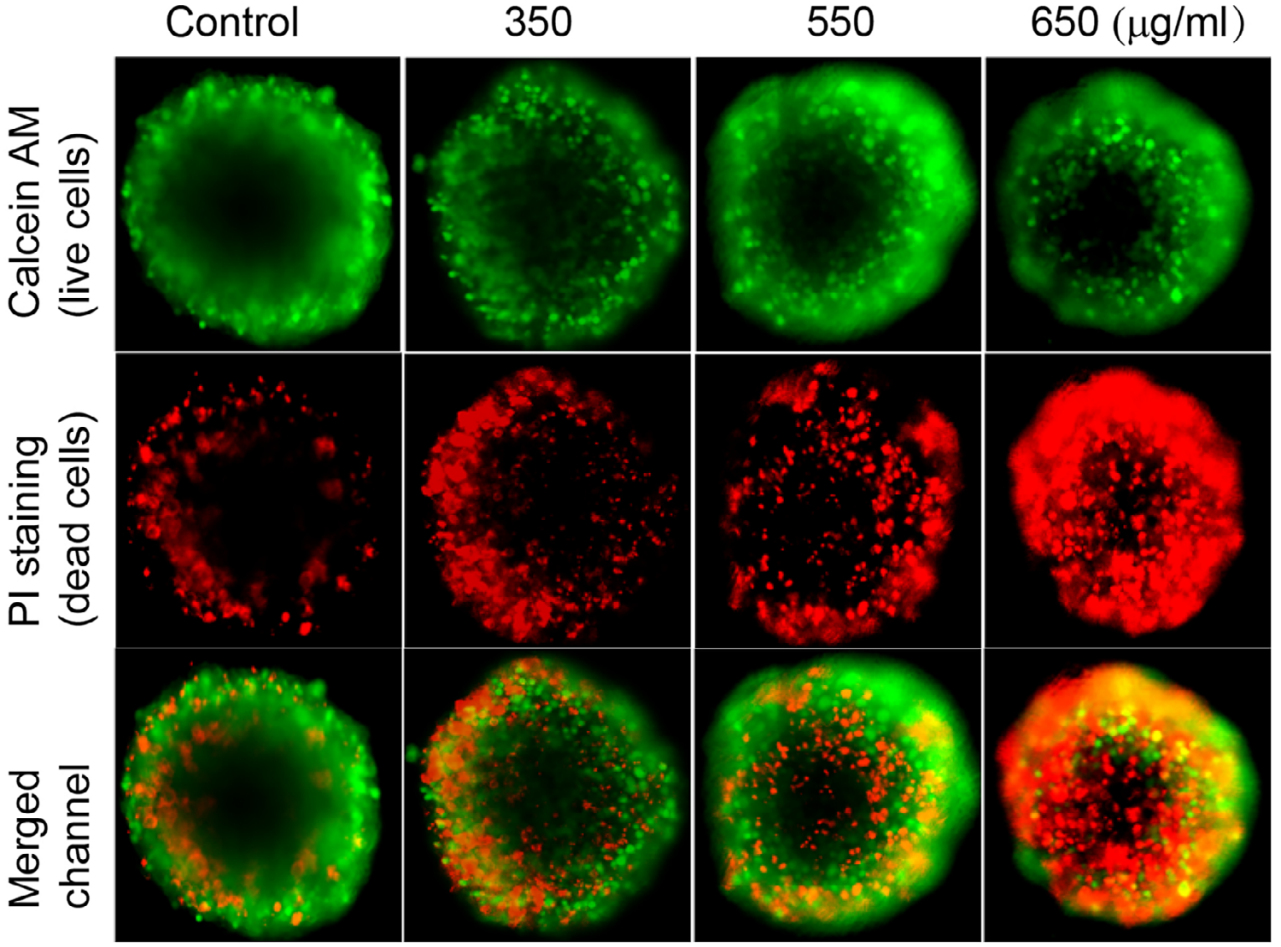
Live and dead staining in 3D spheroids. The green channels represent live cells stained by calcein while the red channel represents the dead cells stained by PI.

As the multi‐doped MoO_x_ nanodots exhibited stronger cytotoxicity toward cancer cells, the study of cytotoxicity and viability analysis was extended to the anti‐proliferative and anti‐cancer study of the multi‐doped MoO_x_ in 3D spheroids formed by HeLa cells.^[^ ^54^ ^]^ The high(NH_4_^+^)‐low(H^+^)MoO_x_ nanodots were added to the spheroids formed in a 96 well ultra‐low attachment (ULA) plate for 24 h and the spheroids were subjected to calcein acetoxymethyl (AM) and propidium iodide (PI) dye to observe the presence of live and dead cells, respectively. The untreated cells were used as the control group. As shown in **Figure** **5**, it was observed that the number of dead cells increased significantly with increasing concentration of high(NH_4_^+^)‐low(H^+^)MoO_x_ from 350–650 µg mL^−1^. The high(NH_4_^+^)‐low(H^+^)MoO_x_ induces effective apoptosis of the majority of cells as seen by the formation of significantly higher number of PI‐stained cells at 650 µg mL^−1^ concentration of high(NH_4_^+^)‐low(H^+^)MoO_x_ nanodot, compared to that of calcein‐stained cells. The concentration of the high(NH_4_^+^)‐low(H^+^)MoO_x_ was varied and the cell death rate was higher along with the elevated concentration, in which the 650 µg mL^−1^ high(NH_4_^+^)‐low(H^+^)MoO_x_ nanodot induced the majority of cell death, and the 350 µg mL^−1^ nanodot sample exhibited the least number of dead cells. In comparison, the untreated control cells maintained ≅ 80% live cells stained by calcein AM.

**Figure 6:**
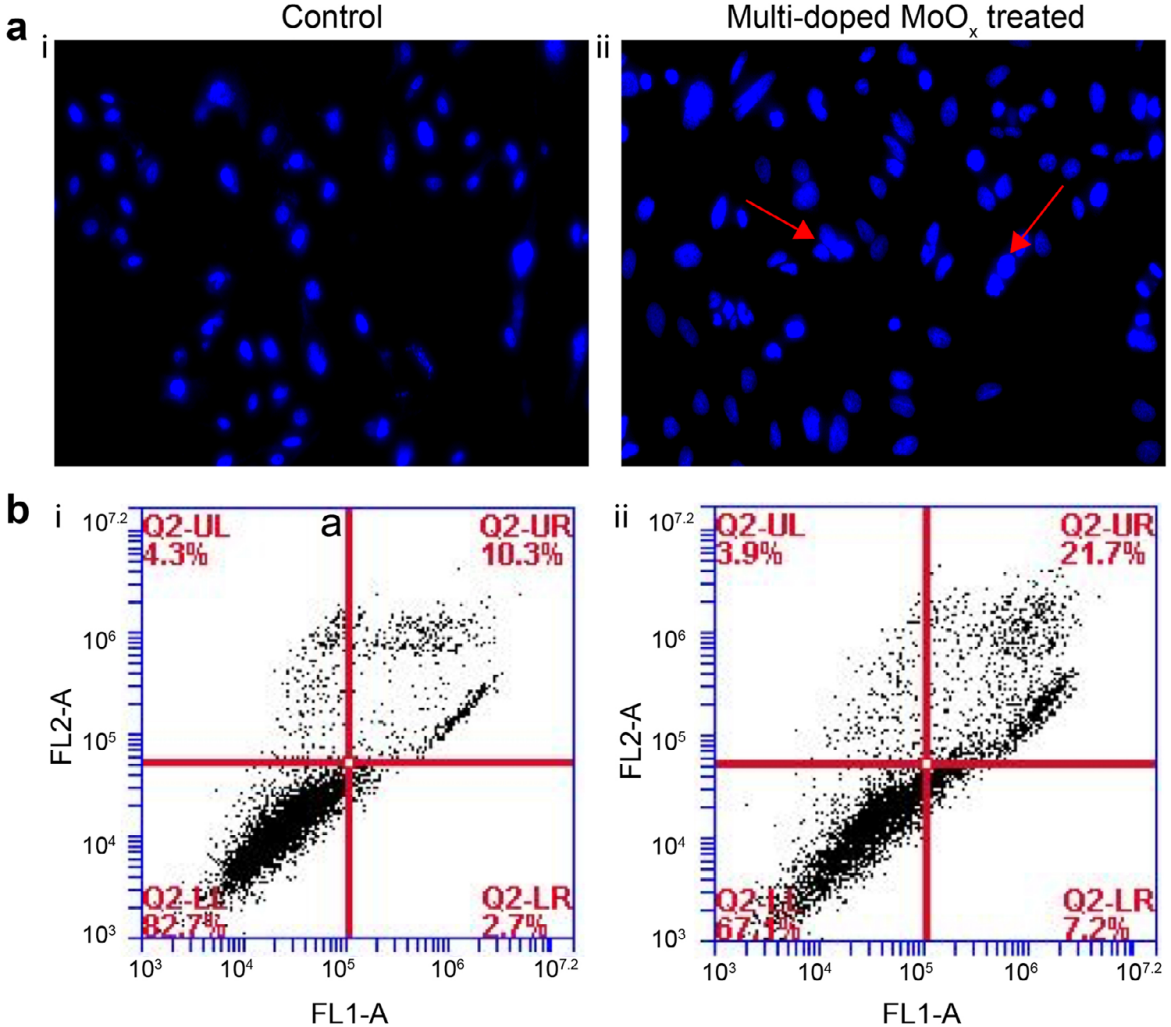
The control of (i) HeLa cell and (ii) high(NH_4_^+^)‐low(H^+^)MoO_x_ nanodot induced apoptosis in HeLa cells by nuclear morphology analysis. b) (i) Non‐treated HeLa cell and (ii) high(NH_4_^+^)‐Low(H^+^)MoO_x_ nanodot treated HeLa cells with induced apoptosis analysis through Annexin‐FITC/PI assay. Annexin V/FITC and PI stained cells were analyzed for the early, late and necrotic apoptosis cells post‐treatment. The 1^st^ (Q2 LL), 2^nd^ (Q2 LR), 3^rd^ (Q2 UR), and 4^th^ (Q2 UL) quadrant represent healthy, early apoptotic, late apoptotic, and necrotic cells, respectively.

The morphological and nuclear changes caused by the multi‐doped MoO_x_ nanodots were observed on the cancer cells with their nucleus stained using Hoechst 33 342^[^ ^55^ ^]^ and observed under a fluorescence microscope for chromatin and nuclear condensation.^[^ ^56^ ^]^ The arrows indicate condensed and fragmented chromatin, suggesting the formation of apoptotic bodies.^[^ ^57^ ^]^ The dye selectively stained the nucleus of both treated and non‐treated HeLa cells. However, the untreated cells did not display strong blue fluorescence, indicative of non‐apoptotic cells. (**Figure** **6**a‐i), and the chromatin and DNA condensation was observed in the high (NH_4_^+^)‐low(H^+^)MoO_x_ treated cells (Figure 6a‐ii) demonstrating the strong cytotoxicity of the nanomaterial. Confirmation of apoptosis in the cells due to the effect of multi‐doped MoO_x_ nanodots was performed by an additional annexin V‐fluorescein isothiocyanate (FITC)/PI staining to both treated and untreated cells. The Annexin assay can help in differentiating between the healthy, early, late and necrotic apoptotic cells. Predominantly, the HeLa cells stained solely with Annexin V‐FITC represent the early apoptotic cell group, whereas the cells stained by both PI and Annexin V‐FITC are considered as the necrotic or late apoptotic cell group. Figure 6b‐i depicts the majority (82.7%) of healthy cells positively stained with Annexin in the 1^st^ quadrant and a relatively low late apoptotic ratio of 10.3% in the 3^rd^ quadrant. In comparison, the ones treated with the high (NH_4_^+^)‐low(H^+^)MoO_x_ showed an over two‐fold late apoptotic rate of 21.7% and early apoptosis rate of 7.2%, as well as a reduced necrotic apoptosis rate of 3.9% and normal rate of 67.1% for HeLa cells (Figure 6b‐ii). The result indicates significant cytotoxicity induction totaling up to 15.6% (counted from the additional rate from the first quadrant) in HeLa cells treated with the multi‐doped MoO_x_ nanodots.

## 3 Conclusion

Unique NH_4_^+^ and H^+^ doped MoO_x_ materials at tunable doping levels have been fabricated through the one‐step hydrothermal method for high performance selective cytotoxicity. Through controlling the matrix of the dual dopants, the high(NH_4_^+^)‐low(H^+^)MoO_x_ nanodot has exhibited favorable band structure, facilitating high efficient ROS production capability even in the dark. The property was demonstrate by fast dark catalysis for organic dye degradation within 20 min comparing with the other two multi‐doped MoO_x_ samples. The working mechanism has been investigated, revealing that the E_VB_ of high(NH_4_^+^)‐low(H^+^)MoO_x_ performs the best in providing necessary oxidizing power for ·OH generation. The material has further demonstrated its strong and selective cytotoxicity toward cervical cancer cell line – HeLa cell, in comparison to another healthy cell line‐ HEK293T. Exposure to multi‐doped MoO_x_ nanodots induced cell death in HeLa and HEK cells through apoptosis, as evidenced by the loss of mitochondrial membrane potential, supported by Hoechst and calcein staining results. While we still agree its future application in biological environments warrants further consideration. Given the ubiquity of water in physiological systems, strategies to maintain a well‐controlled ROS release will be essential. Future studies may combine with other approaches such as surface functionalization, responsive delivery systems, or tumor‐specific triggers to enhance selectivity and minimize off‐target effects. Overall, this work suggests that the ammonium and hydrogen doped MoO_x_ exhibits highly active catalytic behavior. This multi‐doped MoO_x_ also exhibited strong selective toxicity toward cancer cell lines, demonstrating a promising pathway of realizing cancer therapy and may enable advancement in other molecular level therapies.

## 4 Experimental Section

### Synthesis of Multi‐Doped MoO_x_

All samples were synthesized by adding 100 mg ammonium molybdate and 50 mg hydrazine powder in DMF (N,N‐Dimethylmethanamide) and deionized (DI) water solutions with three ratios of 0.2:1, 0.8:1, 1:1. The three solutions were left for stirring at 45 °C for 1.5 h. After that the solutions turn out to be different colors: high(NH_4_^+^)‐high(H^+^)MoO_x_ turned brown, high(NH_4_^+^)‐low(H^+^)MoO_x_ turned Prussian blue and low(NH_4_^+^)‐high(H^+^)MoO_x_ turned blackish color (Figure 1a). Next, the solutions were introduced into autoclaves (Teflon‐lined stainless‐steel) for the purpose of a hydrothermal reaction. These reactions were facilitated at a consistent temperature of 180 °C for an uninterrupted span of 5 h. Post experimentation, the autoclaves were permitted to gradually return to ambient Temperature without external intervention. After the 5 h hydrothermal reaction the three samples altered their colors. The high(NH_4_^+^)‐high(H^+^)MoO_x_ became Prussian blue, high(NH_4_^+^)‐low(H^+^)MoO_x_ color was deep brown and low(NH_4_^+^)‐high(H^+^)MoO_x_ color was light brown. All samples then underwent a cleaning process with DI water, followed by centrifugation, then allowed to dry at ambient temperature.

### Characterization of Structure and Morphology

A series of analyses were conducted on the samples using a variety of specialized equipment. In the acquisition of AFM images, a Bruker Dimension Icon AFM was employed, outfitted with specialized scanning‐assistance software. A JEOL 2100F instrument were utilized for both low‐ and high‐resolution TEM measurements at 200 keV acceleration voltages. A Bruker D8 micro diffractometer was used to capture XRD patterns, with a Vantec 500 detector and a 0.5 mm collimator incorporated. The experimental setup employed a copper source set to 40 kV potential and 40 mA current. Raman spectra were gathered by exciting a laser with a wavelength of 532 nm and a power of 4.5 mW, Using a LabRAM HR Evolution Raman spectrometer from Horiba Scientific. The FTIR spectroscopic measurements were conducted using a PerkinElmer ATR FTIR spectrometer. The Thermo Scientific K‐alpha system and Avantage software was used to carry out XPS. The samples underwent a scanning process using an Al kα monochromated X‐ray source, set to a passing energy of 50 kV with a 50 ms dwell time. For TGA, a Perkin‐Elmer Pyris 1 instrument was used under N_2_ conditions. The heating protocol involved a temperature range of 30 to 600 °C, escalating at a pace of 10 °C per minute. A CHI760D electrochemical workstation containing the standard 3‐electrode cell (NHE) was used for the Mott‐Schottky analysis. The energy of the probe light was fixed at 50 nW and adhered to a power law of 1/3. A Cary 500 spectrometer was employed to carry out UV‐Vis‐NIR spectroscopy. EPR signals were measured with a Bruker EMXplus 9.5/12. The samples were measured with a microwave attenuation of 15 dB and power of 6.32 mW. The sweep width was 100 G centered on 3510 G.

### Assessment of Photocatalytic Performance

The photocatalytic performance of the synthesized samples was tested through the quantification of MB degradation in an aqueous medium. Sixty micrograms of multi‐doped MoO_x_ catalyst was mixed into 50 ml of 500 µm MB solution after dispersal in 3 ml Milli‐Q water. The mixture was stirred in darkness for 20 min to facilitate dye degradation, which was measured using an ultraviolet‐visible spectrophotometer (OCEAN OPTICS, Florida, USA).

### Cell Toxicity Measurement—Cell Culture Condition and Cell Line

The American Type Culture Collection (Manassas, VA) supplied both the HEK293T cells (an embryonic kidney normal cell line) and the human HeLa cells (a cervical cancer cell line). The HEK293T cell lines utilized Dulbecco's Modified Eagle's Medium (DMEM), whereas the HeLa cells were cultured in RPMI 1640 media (GIBCO‐Invitrogen, NY). Both mediums were complemented with 100 µg ml^−1^ of streptomycin, 100 units ml^−1^ of penicillin‐G, 2 mm L‐glutamine and 10% FBS. The incubator environment was kept at 37 °C, composed of 5% CO_2_ and 95% O_2_, in a humidified setting. Fresh medium was replaced every alternative day, and subculturing was performed when cell confluence reached 80–90%. To facilitate further passages, the cells were collected by treating with 0.25% trypsin‐EDTA. All experiments utilized cells from passages 4 to 10. Before introducing multi‐doped MoO_x_ nanodots or silver nanodots for experimental purposes, cells were allowed to adhere to the respective well plates for 24 h. Cells were processed at 24 h post treatment unless otherwise specified.

### Cell Toxicity Measurement—Cell Viability Measurement

The cytotoxicity of the drugs on cell lines was assessed using the MTT assay, conducted 24 h after the treatments. Flat‐bottomed 96‐well plates were used. Cells were seeded at a density of 4 × 10^4^ cells per mL in 96‐well plates with flat bottoms, and left to adhere for 24 h prior to initiating the treatments. Various concentrations of Ag NPs and multi‐doped MoO_x_ nanodots, ranging from 700–5.4 µg mL^−1^, were integrated into the wells, followed by a 24 h incubation period before the execution of the MTT assay. A 5 mg mL^−1^ MTT reagent stock solution was prepared and stored in an environment devoid of light. After thoroughly washing the wells with phosphate‐buffered saline (PBS) (150 mm), 100 µL of the aforementioned stock solution was introduced to each well, followed by a 4 h incubation period for cell interaction with the MTT solution. Subsequently, The insoluble formazan crystals in the live cells were then dissolved using dimethyl sulfoxide (DMSO). The resultant purple solution, reflecting the count of live cells, was analyzed spectrophotometrically at 570 nm, using a Spectramax microplate reader (Plate reader Spectramax Paradigm). Reference wavelength data was also recorded at 630 nm. The calculation of percent growth inhibition was carried out by dividing the mean optical density (OD) of the cells treated with the drug by the mean OD of the vehicle (DMSO), and then multiplying the quotient by 100. Furthermore, Probit software was employed to determine the IC_50_ values.

### Cell Toxicity Measurement—3D Spheroid Assay

Corning Costar Ultra‐Low attachment 24‐well plates were utilized for seeding HeLa cells at a density of 3 × 10^4^ cells mL^−1^. The cells were allowed to adhere and form spheroid‐like structures in complete growth media for a period of 3 days. Following the initial incubation, the spheroids underwent treatment with selected IC_50_ concentrations of multi‐doped MoO_x_ nanodots. The development of the spheroids and the subsequent changes in their diameter were continuously monitored using a phase contrast microscope. After the treatment, PI and calcein AM were introduced to the spheroids to distinguish between live and dead cells. The viable and non‐viable cells within the spheroids were visualized by capturing images through the green and red channels.

### Cell Toxicity Measurement—Morphological Changes Analysis Using Hoechst

Anomalies in nuclear morphology have long been recognized as indicators of apoptosis and cellular stress. To analyze these changes, Hoechst 33 242 was utilized to stain the cells. In this assay, HeLa cells were seeded in 24‐well tissue culture plates at a seeding density of 2 × 10^4^ cells per well. Subsequently, the cells were exposed to selective concentrations, including the IC_50_ concentration of multi‐doped MoO_x_ nanodots. Post treatment, the culture medium was removed, followed by the cells being washed with PBS. A 20 min room temperature fixation process was then performed using 4% paraformaldehyde. Following fixation, the cells were stained with 2 µg mL^−1^ of Hoechst 33 242. Excess dye was removed after a 20‐min incubation period, and the cells were washed three times with cold PBS. Apoptotic cells were then identified by observing the changes in nuclear morphology under blue filters via a ZOE Inverted fluorescent cell imager (Bio‐Rad Laboratories Inc., Hercules, CA, USA).

### Cell Toxicity Measurement—MMP Levels Measurement

In a 24‐well plate, HeLa cells were seeded at a density of 2 × 10^4^ cells per well and adherence was facilitated overnight. Subsequent to this, the cells were subjected to a 24 h treatment with a selected concentration of multi‐doped MoO_x_. Before staining with JC1 dye, a washing process was executed with 150 mm PBS (pH 7.4). JC‐1 stock solution, of a 2 mg mL^−1^ concentration, was prepared to a final concentration of 1 µg mL^−1^ in 150 mm PBS. Subsequently, each well received 500 µL of the JC‐1 dye solution. The plate was then incubated for 20 min at 37 °C in dark environment. HeLa cells were treated with 25 µm carbonyl cyanide m‐CCCP for 30 min at 37 °C to serve as a positive control for mitochondrial depolarization. Following treatment, cells were washed with PBS and stained with JC‐1 dye according to standard protocols for fluorescence imaging. To determine the mitochondrial membrane potential, the relative fluorescence of the green monomeric (depolarized) form at 585 ± 15 nm was measured with excitation at 514 nm, while the red aggregates (hyperpolarized state) form at 590 ± 17.5 nm was measured with excitation at 529 nm. Qualitative images were captured using the red and green filters of a ZOE Inverted fluorescent cell imager.

### Cell Toxicity Measurement—ROS Levels Measurement

ROS levels were associated with the initiation of apoptosis through both intrinsic and extrinsic pathways. To assess ROS levels, A 24‐well plate, initially seeded with 2 × 10^4^ cells per well, was used in this process. A 24 h period was allowed for the cells to adhere undisturbed. Subsequently, the multi‐doped MoO_x_ nanodots were added to the cells and incubated for 24 h. Post incubation, the exhausted medium was removed and replaced with fresh media containing 10 µm carboxy‐DCFDA. DCFDA was added and incubated for 30 min in dark at room temperature. After the incubation, the cells were washed, and fluorescence images of the green channel were captured using a ZOE inverted cell imager. To quantitatively analyze the intensity of the green channel, Image J software was employed.

### Cell Toxicity Measurement—Annexin PI

Apoptosis was assessed by adding Annexin FITC/PI to the cells. In a 12‐well plate, HeLa cells were seeded at a density of 8 × 10^4^ cells per well and adherence was facilitated overnight. Afterward, a 24 h treatment with specific concentrations of multi‐doped MoO_x_ nanodots was administered to the cells. Following the treatment, the cells were washed with PBS and harvested by trypsinization. The resulting cell pellet was then resuspended in Annexin binding buffer containing Annexin‐V‐FITC and PI in a 5:1 ratio. The cell suspension was maintained at a total volume of 15 µL, ensuring coverage of all cells in the tube. A 15‐min room temperature incubation period was followed by the analysis of the cell suspension for red and green fluorescence using the BD Accuri C6 Flow Cytometer (BD Biosciences, San Jose, CA, USA).

## Conflict of Interest

The authors declare no conflict of interest.

## Supporting information



Supporting Information

## Data Availability

The data that support the findings of this study are available in the supplementary material of this article.
