# Supplementary material for: Ultrathin Multi‐Doped Molybdenum Oxide Nanodots as a Tunable Selective Biocatalyst
Source: Adv Sci (Weinh). 2025 Oct 3;12(41):e00643. doi: 10.1002/advs.202500643 (PMC12591189; doi:10.1002/advs.202500643)
Supplement: Supplementary file 1 — Supporting Information [file ADVS-12-e00643-s001.docx]

Supporting Information

Multi-doped molybdenum oxides dots as efficient reactive oxygen species catalysts

*Bao Yue Zhang*, Farjana Haque, Shwathy Ramesan*, Sanjida Afrin, Muhammad Waqas Khan, Haibo Ding, Xin Zhou, Qijie Ma, Jiaru Zhang, Rui Ou, Md Mohiuddin, Enamul Haque, Yichao Wang, Azmira Jannat, Yumin Li, Robi S. Datta, Kate Fox,* Guolang Li, *Hujun Jia, Jian Zhen Ou**

**Table S1** Material composition determination

| Material synthesis condition | Weight loss of H in the form of water (%) | Weight loss of N in the form of NH_4_^+^ (%) | Elemental composition |
| --- | --- | --- | --- |
| In 100% DMF | 7.65 | 3.88 | (NH_4_)_0.36_ H_1.42_MoO_3_  low(NH_4_^+^)-high(H^+^)MoO_x_ |
| In 80% DMF | 5.78 | 8.5 | (NH_4_)_0.81_ H_1.1_MoO_3_  high(NH_4_^+^)-low(H^+^)MoO_x_ |
| In 20% DMF | 7.31 | 6.89 | (NH_4_)_0.66_ H_1.4_MoO_3_  high(NH_4_^+^)-high(H^+^)MoO_x_ |

**Note S1**

Regarding the calculation of free charge carrier concentrations, we consider the typical MoO_3_ crystal with its Space group of *Pnma*, and a = 0.396 nm, b = 1.385 nm and c = 0.369 nm.

Taking (NH_4_)_0.66_ H_1.4_MoO_3_ as an example. Since each NH_4_^+^ and H^+^ will contribute one free electron to the lattice, there are 4 MoO_3_ in each unit cell, the total charge per unit cell is calculated as:

$$0.66\times4+1.4\times4=8.24$$

The volume of each unit cell is:

$$V_{cell}=a\cdot b\cdot c=3.96\times{10}^{-8}cm\times13.86\times{10}^{-8}cm\times3.70\times{10}^{-8}cm$$

$$V_{cell}\approx2.03\times{10}^{-22}{cm}^{3}$$

Number of unit cells in 1 cm^3^:

$$\frac{1}{2.03 \times{10}^{-22}} \approx4.93\times{10}^{21} unit cells per {cm}^{3}$$

Total charge per cm^3^:

$8.24 charge/ unit cell \times4.93\times{10}^{21} unit cells / {cm}^{3}\approx4.06\times{10}^{22}$charge / cm^3^

Similarly, the free charge concentration of (NH_4_)_0.36_ H_1.42_MoO_3_ and (NH_4_)_0.81_ H_1.1_MoO_3_ can be calculated as 3.51 × 10²² cm⁻³ and 3.77 × 10²² cm⁻³, respectively.


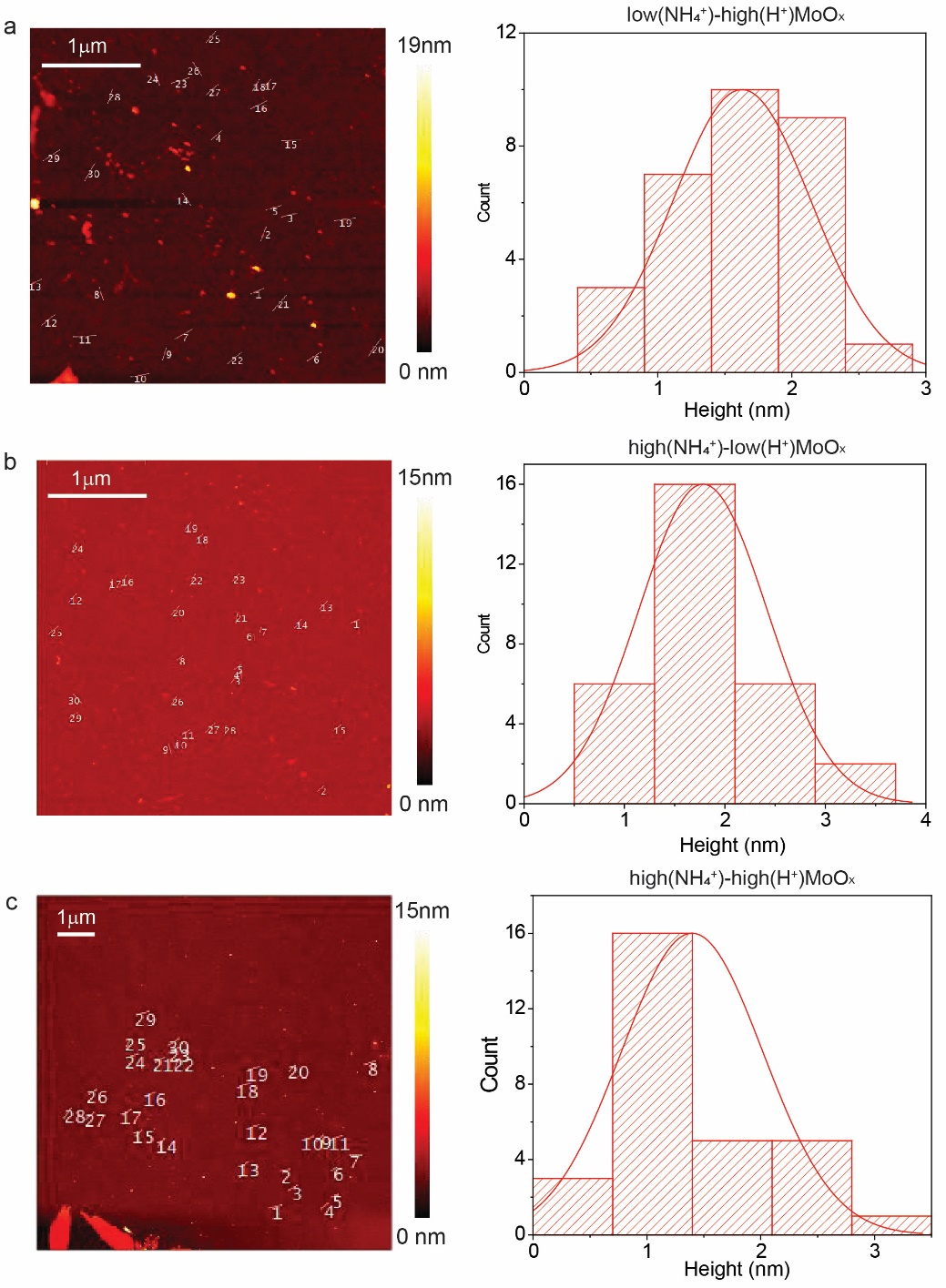


**Figure S1** AFM image (left), histogram plots with normalized distribution curves for the thickness profile of (a) low(NH_4_^+^)-high(H^+^)MoO_x_, (b) high(NH_4_^+^)-low(H^+^)MoO_x_ and (c) high(NH_4_^+^)-high(H^+^)MoO_x_, respectively.


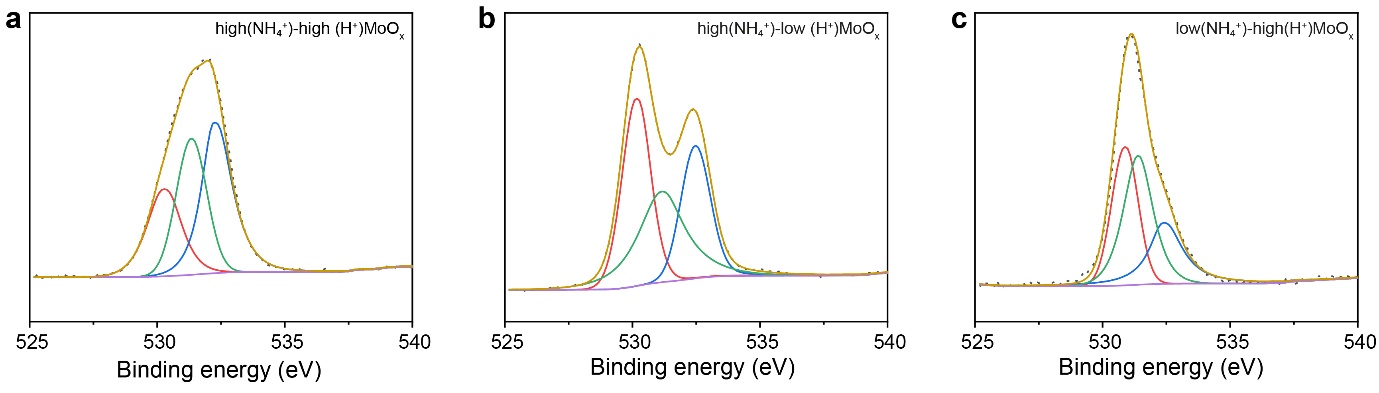


**Figure S2** O1s spectra of (a) high(NH_4_^+^)-high(H^+^)MoO_x_, (b) high(NH_4_^+^)-low(H^+^)MoO_x_ and (c) low(NH_4_^+^)-high(H^+^)MoO_x_.

**
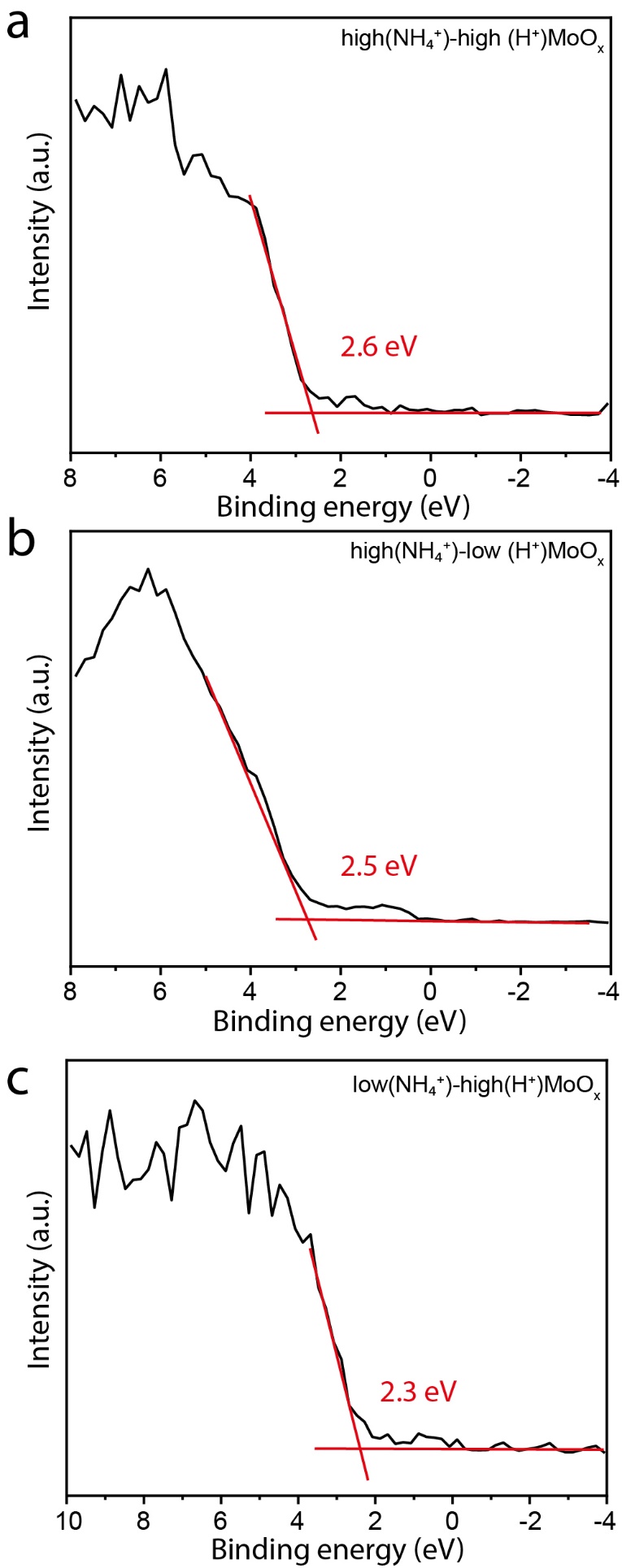
**

**Figure S3** XPS valence spectra of (a) high(NH_4_^+^)-high(H^+^)MoO_x_, (b) high (NH_4_^+^)-low(H^+^)MoO_x_ and (c) low(NH_4_^+^)-high(H^+^)MoO_x_.

**
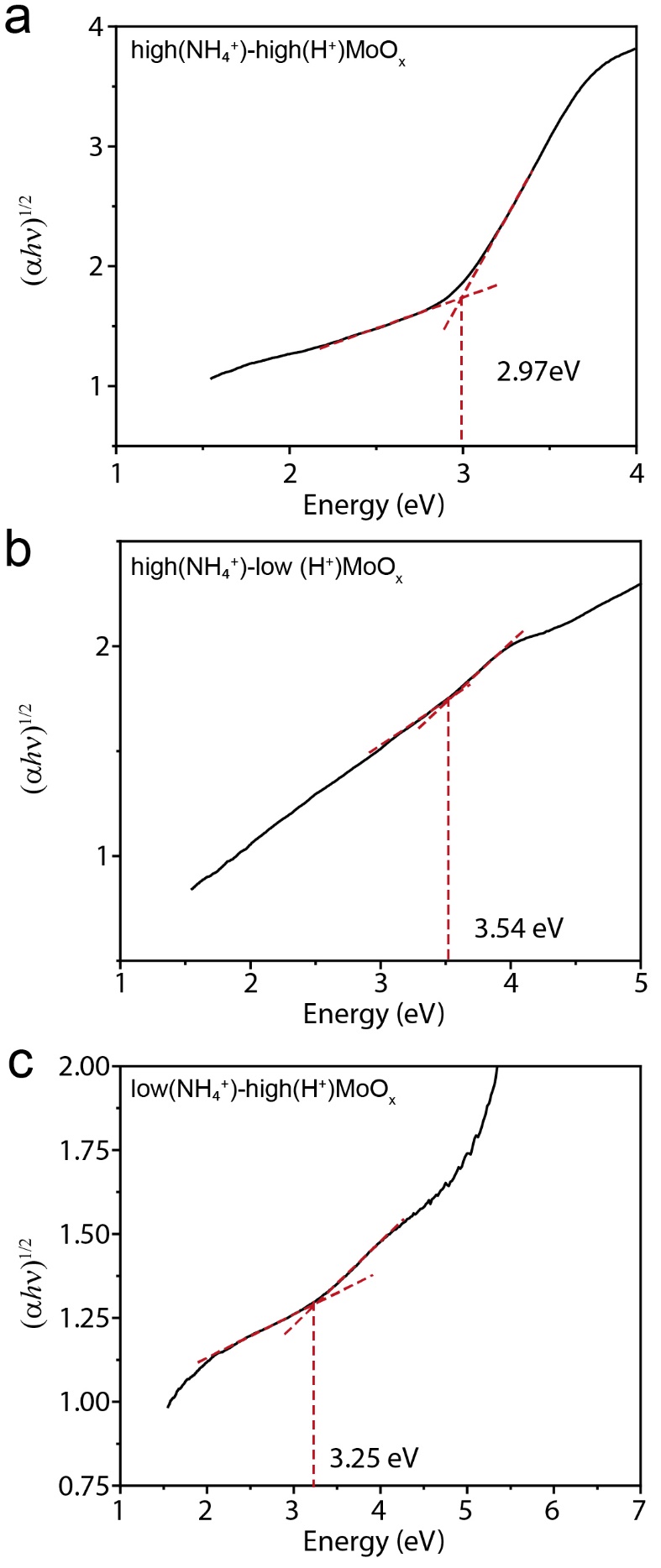
**

**Figure S4** Tauc plot and optical bandgap of (a) high(NH_4_^+^)-high(H^+^)MoO_x_, (b) high (NH_4_^+^)-low(H^+^)MoO_x_ and (c) low(NH_4_^+^)-high(H^+^)MoO_x_.

**
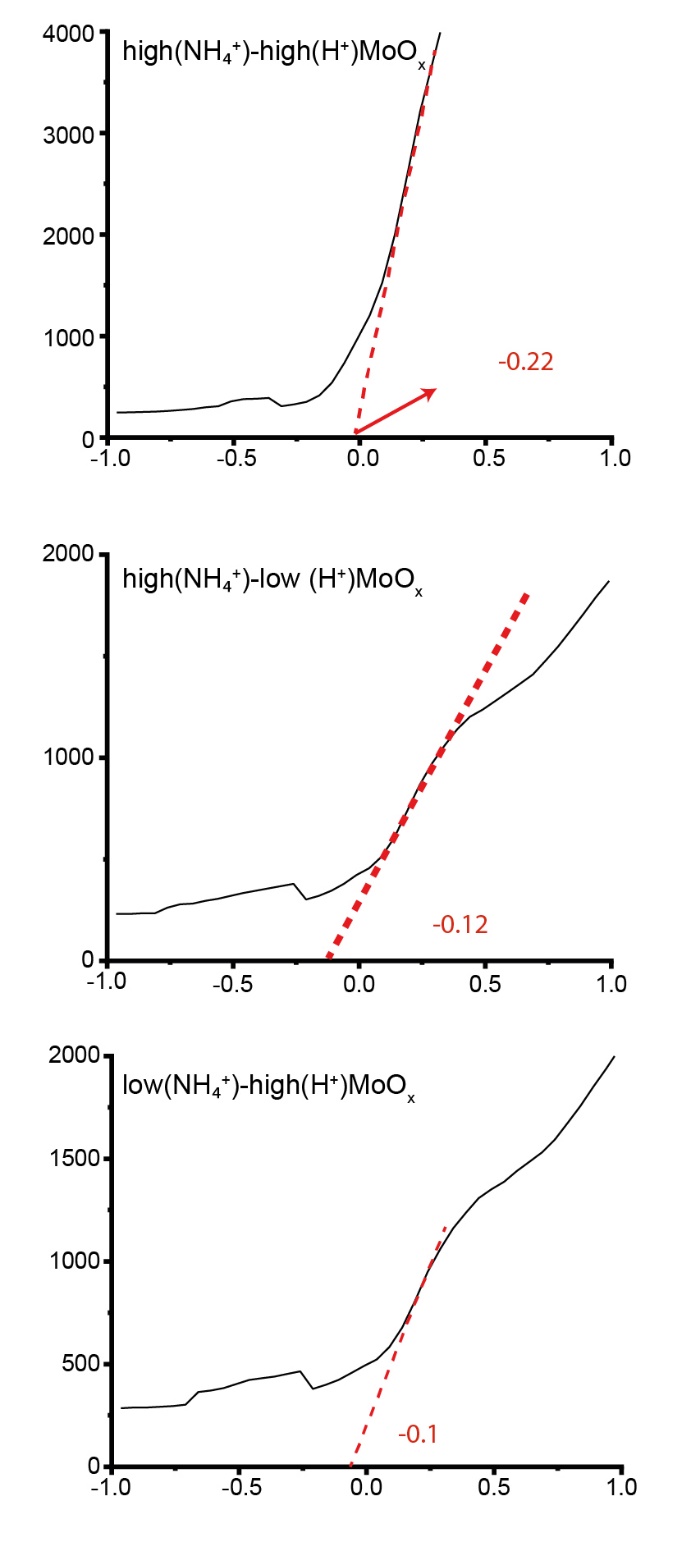
**

**Figure S5** Mott-Schottky plot of (a) high(NH_4_^+^)-high(H^+^)MoO_x_, (b) high (NH_4_^+^)-low(H^+^)MoO_x_ and (c) low(NH_4_^+^)-high(H^+^)MoO_x_.


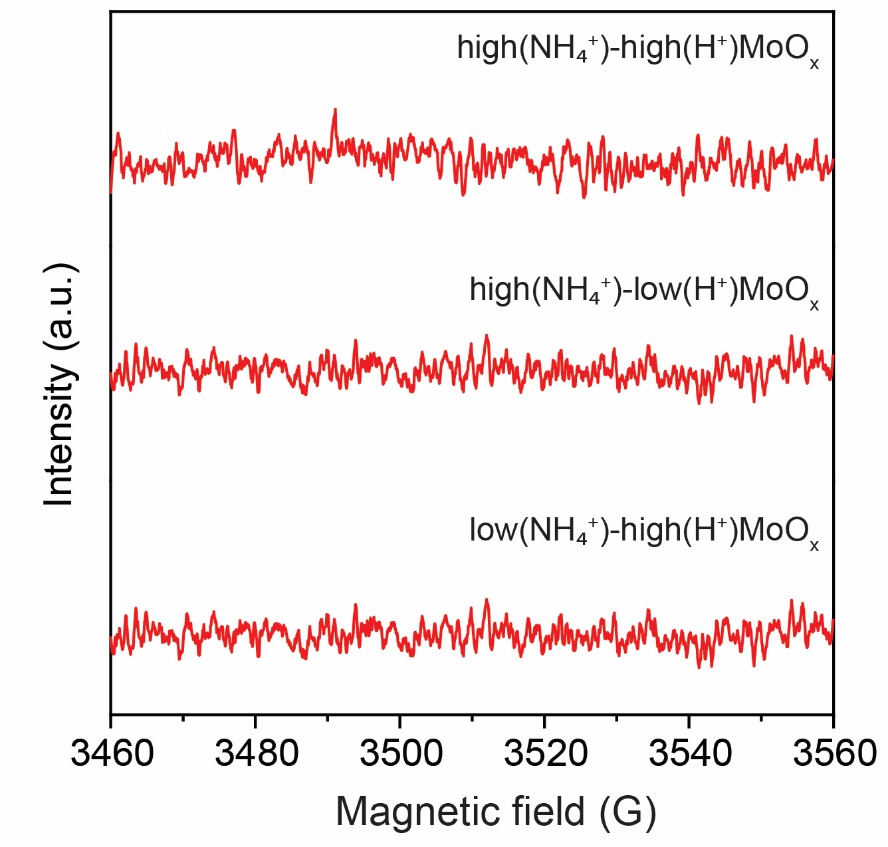


**Figure S6** EPR signals for TEMP - ^1^O_2_ singlet oxygen radicals for three multi-doped MoO_x_.


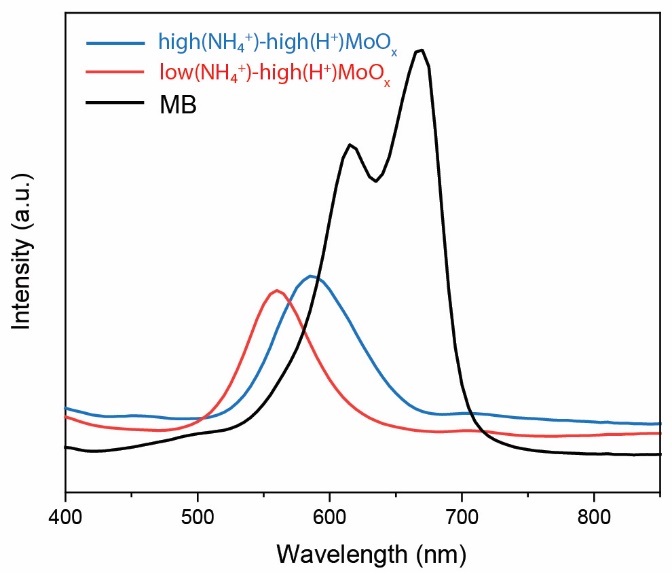


**Figure S7** The UV–Vis absorption spectrum of MB in the presence of 2D high(NH_4_^+^)-high(H^+^)MoO_x_, and low(NH_4_^+^)-high(H^+^)MoO_x_ nanodots tested in room temperature and in dark environment for 20 min.


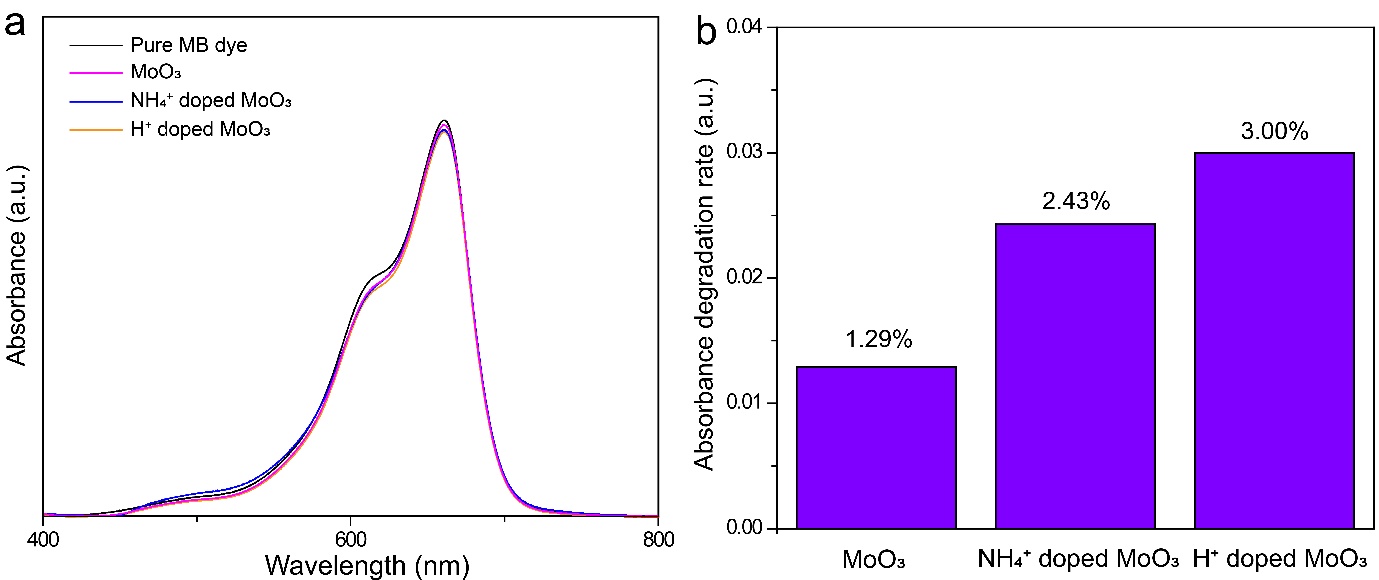


**Figure S8** (a) The baseline subtracted UV-Vis absorption spectrum of MB dye in the presence of the MoO_3_, NH_4_^+^doped MoO_3_, and H^+^ doped MoO_3_ materials at room temperature in the dark after 20 min. (b) Bar chart representing the dye degradation performances of the MoO_3_, NH_4_^+^doped MoO_3_, and H^+^ doped MoO_3_ materials. The absorbance degradation rate is determined by the peak intensity variation in relation to pure MB dye.


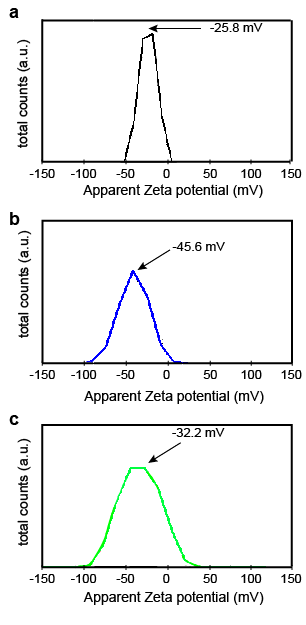


**Figure S9** Zeta potential of (a) high(NH_4_^+^)-high(H^+^)MoO_x_, (b) high (NH_4_^+^)-low(H^+^)MoO_x_ and (c) low(NH_4_^+^)-high(H^+^)MoO_x_ sample.


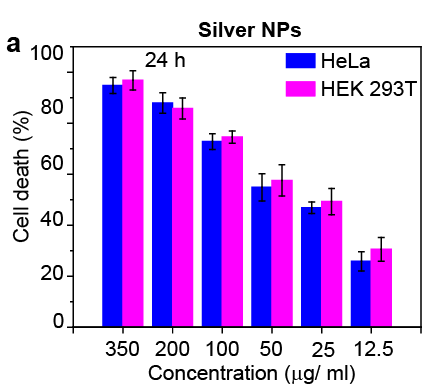


**Figure S10** Graph of MTT assay after 24h at different concentration of silver nanoparticles, showing the rate of proliferation of HeLa cell and HEK 293T cells after exposure to each concentration (12.5 ~350 µg/ml).


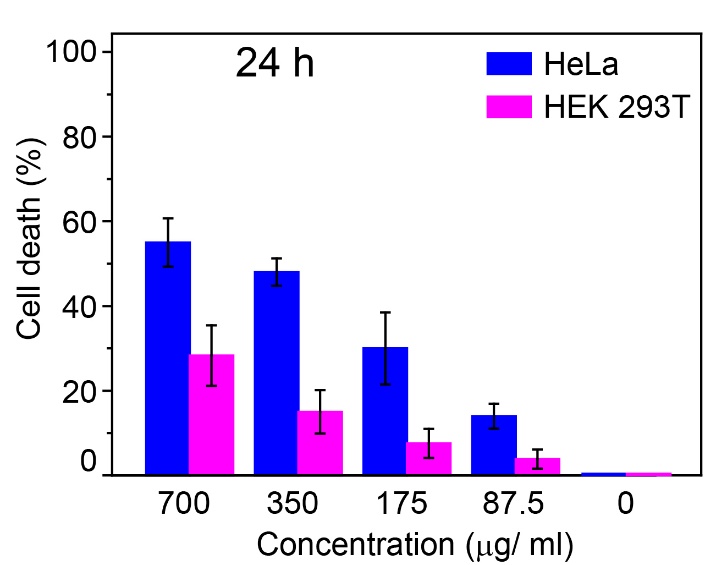


**Figure S11** Graph of MTT assay after 24h at different concentration of high(NH_4_^+^)-low(H^+^)MoO_x_ nanodots, showing the rate of proliferation of HeLa cell and HEK 293T cells after exposure to each concentration (87.5 ~700 µg/ml).


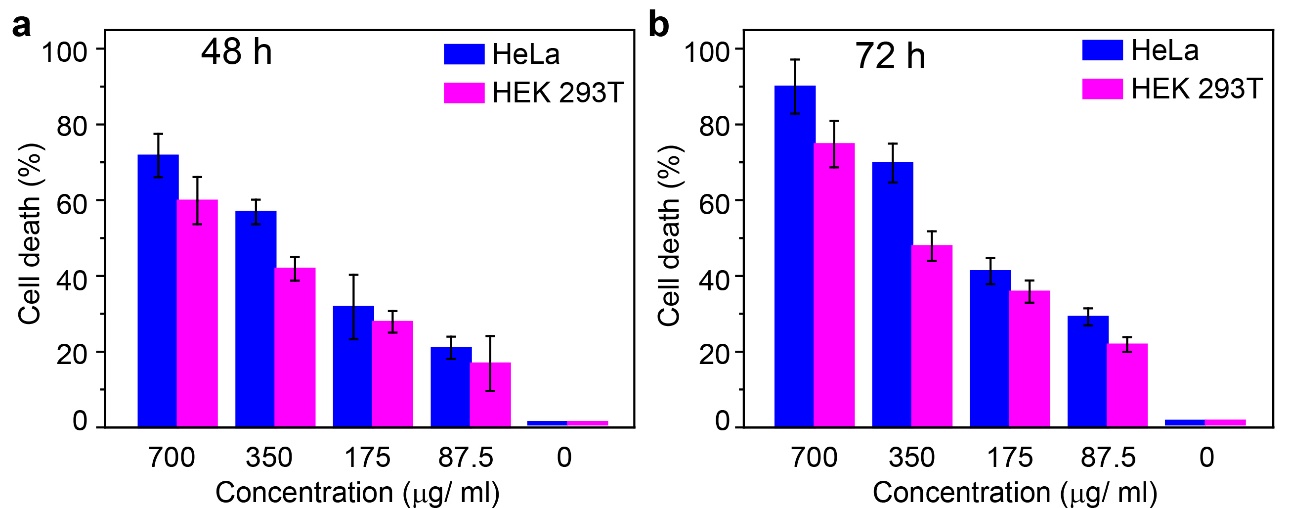


**Figure S12** Graph of MTT assay after (a) 48h and (b) 72h at different concentration of high(NH_4_^+^)-low(H^+^)MoO_x_ nanodots, showing the rate of proliferation of HeLa cell and HEK 293T cells after exposure to each concentration (87.5 ~700 µg/ml).
